# Co-Application of Tetramycin and Matrine Improves Resistance of Kiwifruit against Soft Rot Disease and Enhances Its Quality and Amino Acids

**DOI:** 10.3390/antibiotics11050671

**Published:** 2022-05-17

**Authors:** Cheng Zhang, Wenzhi Li, Youhua Long, Yue Su, Qinghai Zhang

**Affiliations:** 1 Key Laboratory of Environmental Pollution Monitoring and Disease of Ministry of Education, School of Public Health, Guizhou Medical University, Guiyang 550025, China; chengz76@gmc.edu.cn; 2 Research Center for Engineering Technology of Kiwifruit, Institute of Crop Protection, Teaching Experimental Farm, College of Agriculture, Guizhou University, Guiyang 550025, China; lwz9512@126.com (W.L.); yhlong3@gzu.edu.cn (Y.L.); 3 Department of Agricultural Engineering, Guizhou Vocational College of Agriculture, Qingzhen 551400, China

**Keywords:** kiwifruit, tetramycin, matrine, *Botryosphaeria dothidea*, *Phomopsis* sp., soft rot disease

## Abstract

Soft rot disease caused by *Botryosphaeria dothidea* and *Phomopsis* sp. is the most serious fungal disease of the kiwifruit production area in southwest China. In this work, the role of the co-application of tetramycin and matrine in the resistance of kiwifruit fruits against soft rot disease and its effects on development, quality and amino acids of kiwifruit fruits were investigated. The results indicate that matrine exhibited an outstanding toxicity against *B. dothidea* RF-1 and *Phomopsis* sp. RF-2 with EC_50_ values of 0.442 and 0.332 mg kg^−1^. The foliar co-application of 0.3% tetramycin aqueous solutions (AS) 5000-fold liquid + 0.5% matrine AS 1000-fold liquid could effectively control soft rot disease with a control efficacy of 82.68%, which was significantly (*p* < 0.05) higher than 75.19% of 0.3% tetramycin AS 5000-fold liquid and significantly (*p* < 0.01) higher than 68.50% of 0.5% matrine AS 500-fold liquid. Moreover, the co-application of tetramycin and matrine was more effective than tetramycin or matrine alone in improving disease resistance, quality and amino acids of kiwifruit fruits. This study highlights that the co-application of tetramycin and matrine can be used as a practicable, cost-effective and environmentally friendly candidate or alternative approach for controlling soft rot disease of kiwifruit.

## 1. Introduction

As a typical third-generation fruit, kiwifruit (*Actinidia chinensis*) has high nutritional, medicinal and economic value due to its richness in vitamin C, essential amino acids and various minerals [1,2]. Recently, the kiwifruit industry in China has been rapidly developed, especially in the Guizhou Province of Southwest China, where the planting area has reached over 40,000 ha [2,3]. Nonetheless, soft rot disease caused by fungal pathogens such as *Botryosphaeria dothidea*, *Phomopsis* sp., *Cryptosporiopsis actinidiae*, *Botrytis cinerea*, *Cylindrocarpon* sp. and *Phoma exigua*, etc., is a serious disease of postharvest kiwifruit, and affects its quality, yield and economic value [4,5,6,7,8,9,10,11]. Our previous studies found that soft rot disease caused by *B. dothidea* and *Phomopsis* sp. was the most serious fungal disease of kiwifruit production area in Guizhou Province, of which infection peak periods were from May 20 to June 13 and August 2 to August 12 [9,10,11]. *B. dothidea* and *Phomopsis* sp. can enter kiwifruit tissue at early growth stages and remain latent in the tissues until fruit ripeness, thus triggering fruit rot symptoms during storage, as well as causing 30~50% economic losses [11,12]. Consequently, the excogitation of the practicable, alternative, cost-effective and environmentally friendly strategies for controlling soft rot disease of kiwifruit is an eternal research hotspot.

Considering chemical fungicide residue and pathogen resistance, natural products that are mild and basically harmless are preferred by consumers and are increasingly used as popular alternatives or complementary approaches for plant disease management in agriculture [13,14]. Meanwhile, a single natural product is often less effective in controlling plant diseases compared to chemical fungicides. Synergistic application of natural products not only extends their antimicrobial spectrum, reduces the amount of fungicide and decreases control cost, it also plays a key role in overcoming and delaying the fungicide resistance of pathogens [15]. For instance, Li et al. [16] reported that co-application of allicin and chitosan increased resistance of *Rosa roxburghii* against powdery mildew and enhanced its yield and quality. Wang et al. [17] reported that chitosan used together with isopyrazam–azoxystrobin could effectively control leaf spot disease of kiwifruit and reduce isopyrazam–azoxystrobin application. In our previous study, we found that chitosan is a practicable adjuvant of tetramycin for controlling leaf spot disease of kiwifruit, enhancing resistance and photosynthesis of kiwifruit leaves, and improving quality and amino acids of kiwifruit fruits [3]. Accordingly, synergistic application of appropriate natural products against soft rot disease of kiwifruit is worth further exploration and development.

**Figure 1:**
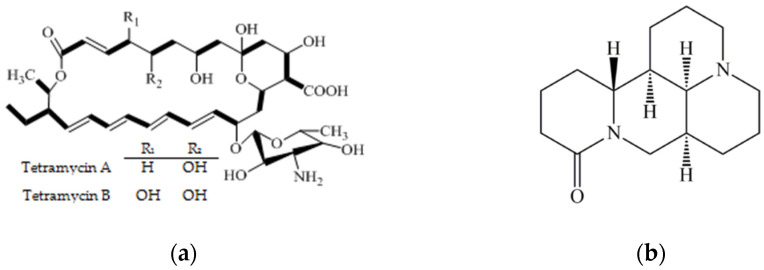
The chemical structures of tetramycin (**a**) and matrine (**b**).

Tetramycin (Figure 1a), the metabolites of *Streptomyces hygrospinosus* var. beijingensis, is a novel medical, agricultural and natural antibiotic that contains two active components (tetramycin A and tetramycin B) [18,19]. In agriculture, tetramycin had been demonstrated to possess outstanding bioactivity against many plant–pathogenic fungi, including *Botrytis cinerea*, *Pyricularia oryzae*, *Colletotrichum scovillei*, *Passalora fulva* and *Phytophthora capsici*, etc. [19,20,21,22,23,24]. It is gradually becoming a preferred alternative or complement to chemical fungicides or conventional antibiotics due to its promising antimicrobial activities and eco-friendly advantages, and it is already registered for controlling fruit, vegetable and rice diseases [19,25,26]. In our previous study, Wang et al. [27] found that tetramycin exhibited superior antimicrobial activity against various kiwifruit pathogens, such as *Pseudomonas syringae* pv. *actinidiae*, *Botryosphaeria dothidea*, *Phomopsis* sp., *Pseudomonas fulva*, *Alternaria tenuissima*, *Agrobacterium tumefaciens*, *Armillariella mellea* and *Phytophthora cactorum*. In a subsequent study, we also found that chitosan could effectively enhance tetramycin against soft rot of kiwifruit, and their co-application was more effective than tetramycin or chitosan alone in enhancing disease resistance, growth, quality and aroma of kiwifruit [12]. Considering the severity of soft rot disease in kiwifruit, it is of great practical significance to establish various candidate, practicable and environmentally friendly approaches and find various adjuvants of tetracycin.

Matrine (Figure 1b), a natural tetracyclo-quinolizindine alkaloid, is a bioactive compound of Chinese herbs, including *Sophora flavescens* and *Radix Sophorae tonkinensis*, and has been widely used in medicine and agriculture fields [28]. In medicine, a large number of evidence has suggested that matrine possesses anti-cancer, anti-inflammatory, anti-oxidant, antiviral, antimicrobial, anti-fibrotic, anti-allergic, antinociceptive, hepatoprotective, cardioprotective, and neuroprotective properties [29,30]. In agriculture, matrine has also been verified to have satisfactory antiviral, fungicidal and insecticidal activities [31,32,33,34]. For instance, Fu et al. [34] reported that matrine exhibited broad-spectrum antifungal activity, such as against *Cochliobolus miyabeanus*, *Rhizoctonia solani*, *Fusarium moniliforme*, *Alternaria brassicae*. Sun et al. [35] reported that combined matrine and osthole had a good effect and could be used as an alternative fungicide against Sorghum purple spot disease caused by *Cercospora sorghi*. In China, matrine has been widely registered and used for controlling the pests and diseases of fruits, vegetables, crops, etc. However, to date, there is little to no attention or documentation available regarding the application of matrine for controlling soft rot disease of kiwifruit. Meanwhile, whether matrine can be used as an effective adjuvant to promote tetramycin against soft rot disease of kiwifruit is worth further attention.

In this work, the bioactivities of various botanical fungicides against *B. dothidea* and *Phomopsis* sp. were first determined. Subsequently, the control efficacy of the combined application of tetramycin and matrine against soft rot disease of kiwifruit was evaluated. Moreover, the effects of the combined application of tetramycin and matrine on disease resistance, development, quality and amino acids of kiwifruit fruits were investigated. These findings provide a practicable, green, and safe candidate approach for controlling soft rot disease of kiwifruit.

## 2. Materials and Methods

### 2.1. Pathogens, Fungicides and Culture Medium

Highly pathogenici pathogens (*Botryosphaeria dothidea* RF-1 and *Phomopsis* sp. RF-2) were provided by the Research Center for Engineering Technology of Kiwifruit, Guizhou University, Guiyang City, Guizhou Province, China. The information of tested fungicides is shown in Table 1. Potato dextrose agar (PDA, 200 g of potato, 20 g of dextrose, 15 g of agar and 1000 mL of distilled water) medium was sterilized at 121 °C for 30 min, and its pH value was neutral.

### 2.2. Field Control Experiment Site

Field control experiments of soft rot disease by tetramycin + matrine, tetramycin, and matrine were conducted in an orchard of kiwifruit with a 7-year-old ‘Guichang’ cultivar in Ganba Village, Longchang Town, Xiuwen Country, Guiyang City, Guizhou Province, China (26°79′80″ N, 106°56′58″ E). Kiwifruit trees were carried by the concrete ‘T’ type frames, and their planting density was 74 plants per 666.7 m^2^, with plant spacing at 3.00 × 3.00 m. Male plants accounted for 1/9 of the total plants. The annual rainfall, mean altitude, annual sunshine duration, frostless season and mean temperature of the kiwifruit orchard was about 1, 293 mm, 1, 300 m, 1, 139.2 hours, 266 days and 13.2~15 °C, respectively. The physical and chemical characteristics of planting soils in the kiwifruit orchard are shown in Table 2.

### 2.3. In Vitro Toxicity Tests of Tetramycin and Botanical Fungicides

The in vitro toxicities of tetramycin or botanical fungicides against *B. dothidea* RF-1 and *Phomopsis* sp. RF-2 were tested by the mycelium growth rate method [3]. The tested solutions of tetramycin or botanical fungicides at five gradient levels, which were set based on the pre-experiment results, were prepared with sterilized water. Then, 1 mL tested solution was uniformly mixed into 9 mL sterilized PDA solution (45~55 °C), and 1 mL sterilized water was used as control solution. Then, the mixed fungicide–PDA solution was transferred to 90 mm diameter petri dishes. Then, it stood until solidification to prepare a fungicide-containing PDA. Subsequently, a 5 mm diameter *B. dothidea* RF-1 or *Phomopsis* sp. RF-2 disc was cut from the active growth site of a 7-day-old *B. dothidea* RF-1 or *Phomopsis* sp. RF-2 cultured on a PDA plate without fungicide, and it was placed in the fungicide-containing PDA plate center with the inoculum side down. Each tested solution was set at three replicates. After the treated plates was cultured at 28 °C until the mycelium growth was almost covered the control plates, the mycelium diameters of *B. dothidea* RF-1 or *Phomopsis* sp. RF-2 growth in the fungicide-containing PDA plates were determined by criss-cross method. Five inhibition rates of each fungicide under five gradient concentrations against *B. dothidea* RF-1 or *Phomopsis* sp. RF-2 were calculated, respectively. Finally, the linear regression equation and EC_50_ (effective concentration of 50% inhibition rate) values of tetramycin or botanical fungicides against *B. dothidea* RF-1 or *Phomopsis* sp. RF-2 were obtained according to five series concentrations of fungicides and their corresponding inhibition rates. The inhibition rate was calculated as Equation (1):

Inhibition rate (%) = 100 × ((Mycelium diameter of control-Mycelium diameter of fungicide)/(Mycelium diameter of control-5))(1)

### 2.4. Field Control Experiment of Soft Rot Disease of Kiwifruit

According to the results of in vitro toxicity tests of tetramycin and botanical fungicides against pathogens of soft rot disease, tetramycin and matrine were preferred as the field control fungicides. A field control experiment was set for four experimental treatments: 0.3% tetramycin AS 5000-fold + 0.5% matrine AS 1000-fold dilution liquid, 0.3% tetramycin AS 5000-fold dilution liquid, 0.5% matrine AS 500-fold dilution liquid, and clear water (control). Completely randomized experimental design and foliar spray method were used for the field control experiment of leaf spot disease. Each treatment was set at three replicates, and a total of twelve plots with three repetitions were arranged randomly. Each plot contained nine trees, and five trees on the diagonal were used for investigation. Based on our previous study, from May 20 to June 13 and August 2 to August 12 were infection periods of soft rot pathogens on ‘Guichang’ kiwifruit [11]. Accordingly, on May 19 and August 1, about 1500 and 2000 mL of fungicide dilution liquid was sprayed onto each kiwifruit plant (including fruits, leaves, buds and stems).

### 2.5. Investigation of Control Effect of Soft Rot Disease in Kiwifruit Fruits

A total of 250 fruits was randomly collected from the middle, east, south, west and north parts of five trees in each plot on September 2. Of these, 150 fruits were used for investigating soft rot disease, and another 100 fruits was used for investigating resistance, growth, quality and amino acids. When fruits reached an edible state, their soft rot disease was investigated. The incidence rate and control effect of soft rot disease were respectively calculated as Equations (2) and (3):

Incidence rate (%) = (Number of diseased kiwifruit/Total number of kiwifruit) × 100(2)

Control effect (%) = ((Incidence rate in control-Incidence rate in treatment)/Incidence rate of control) × 100(3)

### 2.6. Investigation of Resistance, Growth, Quality and Amino Acids of Kiwifruit Fruits

When fruits reached an edible state, their disease resistance parameters including total phenolics, total flavonoids, soluble protein and malonaldehyde (MDA), as well as superoxide dismutase (SOD), peroxidase (POD), polyphenoloxidase (PPO) and phenylalanine ammonia lyase (PAL) activities were measured according to Zhang et al. [9,10] and Wang et al. [12]. The longitudinal, transverse and lateral diameters, as well as fruit shape index, single fruit volume and weight of fruits were measured as described by Zhang et al. [3]. Meanwhile, the vitamin C, soluble solid, soluble sugar, titratable acidity and dry matter of fruits were also measured according to Zhang et al. [10]. Simultaneously, the contents of 17 hydrolyzed amino acids in the fruits were measured using a High Performance Liquid Chromatography (HPLC) system (ThermoFisher U3000). Furthermore, sweet, flavor, bitter, aromatic, essential, nonessential and total amino acids were counted [36].

### 2.7. Statistical Analyses

All data are exhibited as the mean ± standard deviation (SD) of three replicate results. SPSS 18.0 (SPSS Inc., Chicago, IL, USA) was used for calculating the regression equation and EC_50_ values, analyzing variance and normality of data. A one-way analysis of variance (ANOVA) and quantile–quantile (Q–Q) plot test were determined for the difference significances and normality of data, respectively. Origin 10.0 was used to draw charts.

## 3. Results

### 3.1. Toxicity of Tetramycin and Botanical Fungicides against Soft Rot Pathogens

The toxicity of tetramycin and botanical fungicides against *B. dothidea* RF-1 and *Phomopsis* sp. RF-2. of soft rot disease is shown in Table 3. First, 0.3% Tetramycin AS exhibited an excellent toxicity against *B. dothidea* RF-1 and *Phomopsis* sp. RF-2 with EC_50_ values of 0.143 and 0.094 mg kg^−1^, which were higher by 4.76 and 3.20, 131.13 and 274.97, 592.62 and 409.80, 656.78 and 608.56, and 9525.24 and 2605.62 folds compared to 0.3% eugenol SL, 1.0% osthole EW, 80% ethylicin EC, 0.5% physcion AS and 0.5% berberine AS, respectively. Meanwhile, 0.5% matrine AS also had an outstanding toxicity against *B. dothidea* RF-1 and *Phomopsis* sp. RF-2 with EC_50_ values of 0.442 and 0.332 mg kg^−1^, which were higher by 42.43 and 77.85, 191.73 and 116.03, 212.49 and 172.30, and 3081.70 and 737.73 folds compared to 1.0% osthole EW, 80% ethylicin EC, 0.5% physcion AS and 0.5% berberine AS, respectively. The results indicate that 0.3% tetramycin AS and 0.5% matrine AS had a notable potential for controlling soft rot disease of kiwifruit in the field. Although 0.3% eugenol SL also had a relatively superior toxicity against *B. dothidea* RF-1 and *Phomopsis* sp. RF-2, its EC_50_ value to the dominant pathogen *B. dothidea* was lower than that of 0.5% matrine AS. Moreover, 0.3% tetramycin AS and 0.5% matrine AS had a same dosage form; hence, they were optimized as the field control fungicides of soft rot disease in kiwifruit.

### 3.2. Control Effects of Tetramycin and Matrine against Soft Rot Disease of Kiwifruit

The control effects of tetramycin + matrine, tetramycin alone and matrine alone against soft rot disease of kiwifruit are shown in Table 4. Tetramycin + matrine, tetramycin and matrine significantly (*p* < 0.01) decreased the incidence rate of soft rot disease of kiwifruit, and tetramycin + matrine was the most effective. The control effect of tetramycin + matrine against soft rot disease was 82.68%, which was significant (*p* < 0.05) higher than 75.19% of tetramycin and was significantly (*p* < 0.01) higher than 68.50% of matrine. The results demonstrate that matrine used together with tetramycin effectively controlled soft rot disease of kiwifruit, whose control effect was superior to that of tetramycin or matrine alone.

### 3.3. Effects of Tetramycin and Matrine on Resistance Parameters of Kiwifruit Fruits

**Figure 2:**
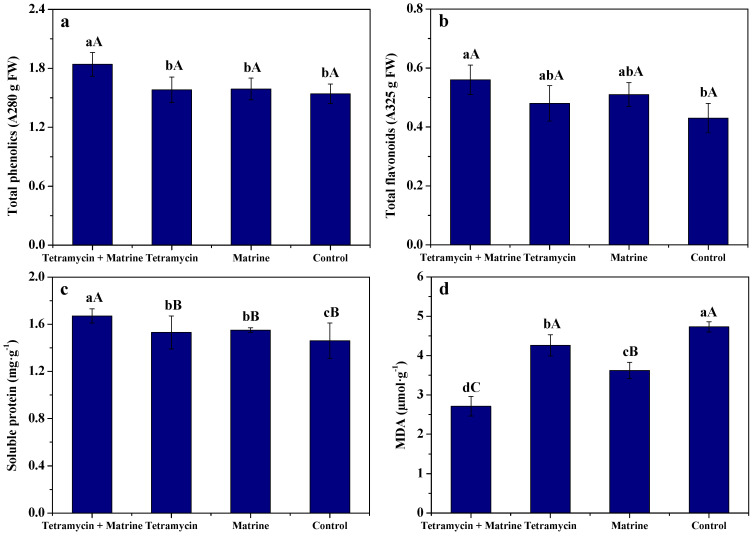
The effects of tetramycin and matrine on the contents of phenolics (**a**), flavonoids (**b**), soluble protein (**c**), and MDA (**d**) in kiwifruit fruits. Values indicate the mean ± SD (*n* = 3). Different capital and small letters show significant differences at 1% (*p* < 0.01) and 5% (*p* < 0.05) levels, respectively.

Figure 2 depicts the effects of tetramycin + matrine, tetramycin and matrine on the total phenolics, total flavonoids, soluble protein, and MDA of kiwifruit fruits. Compared to tetramycin, matrine or control, and tetramycin + matrine significantly (*p* < 0.05) increased the contents of total phenolics and total flavonoids of kiwifruit fruits and significantly (*p* < 0.01) increased their soluble protein content, as well as significantly (*p* < 0.01) reduced their MDA content. Total phenolics and total flavonoids of kiwifruit fruits treated by tetramycin or matrine alone had no significant (*p* < 0.05) difference to those of the control, while their soluble protein content was significantly (*p* < 0.05) higher than that of the control. Moreover, MDA of kiwifruit fruits treated by tetramycin or matrine alone was significant (*p* < 0.01) or significantly (*p* < 0.05) higher than that of the control, respectively. These results demonstrate that matrine used together with tetramycin enhanced total phenolics, total flavonoids and soluble protein contents of kiwifruit fruits and inhibited their MDA content.

**Figure 3:**
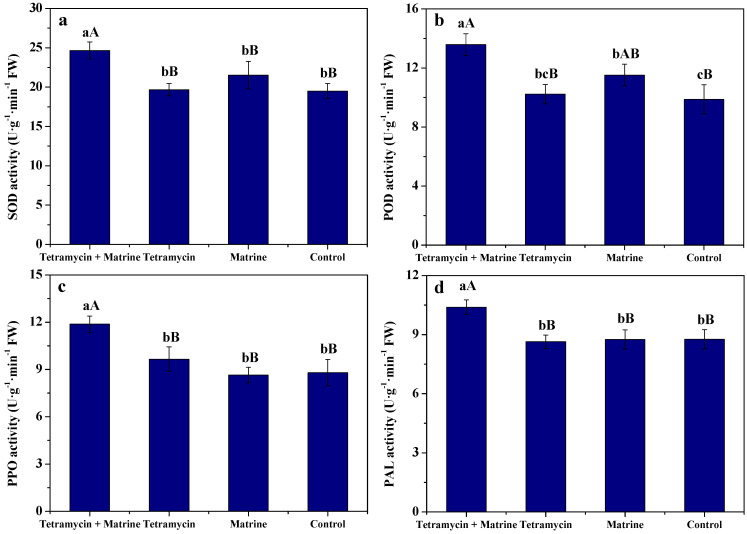
The effects of tetramycin and matrine on SOD (**a**), POD (**b**), PPO (**c**), and PAL (**d**) activities of kiwifruit fruits. Values indicate the mean ± SD (*n* = 3). Different capital and small letters show significant differences at 1% (*p* < 0.01) and 5% (*p* < 0.05) levels, respectively.

Figure 3 depicts the effects of tetramycin + matrine, tetramycin and matrine on SOD, POD, PPO, and PAL activities of kiwifruit fruits. Compared to tetramycin, matrine or control, tetramycin + matrine significantly (*p* < 0.01) enhanced SOD, PPO, and PAL activities of kiwifruit fruits. Compared to tetramycin or control, tetramycin + matrine also significantly (*p* < 0.01) enhanced POD activity of kiwifruit fruits; meanwhile, POD activity of kiwifruit fruits treated by tetramycin + matrine was significantly (*p* < 0.05) higher than that of matrine, while that of matrine was also significantly (*p* < 0.05) higher than that of the control. SOD, PPO, and PAL activities of kiwifruit fruits treated by tetramycin or matrine alone had no significant (*p* < 0.05) difference to those of the control; their POD activity treated by tetramycin also had no significant difference to that of the control. These findings demonstrate that matrine used together with tetramycin notably improved SOD, POD, PPO, and PAL activities of kiwifruit fruits.

### 3.4. Effects of Tetramycin and Matrine on Growth and Quality of Kiwifruit Fruits

The effects of tetramycin + matrine, tetramycin, and matrine on the development of kiwifruit fruits are shown in Table 5. Longitudinal, transverse, lateral diameters and fruit shape index of fruits showed no significant (*p* < 0.05) differences among the four treatments. Compared to control, tetramycin + matrine could significantly (*p* < 0.05) enhance the single fruit volume and weight of kiwifruit fruits, but there was no significant (*p* < 0.05) difference to those of kiwifruit fruits treated by tetramycin. Meanwhile, the single fruit weight of kiwifruit fruits treated by tetramycin + matrine was significantly (*p* < 0.05) higher than those of matrine. The results reveal that matrine used together with tetramycin effectively promoted fruit development and yield formation of kiwifruit.

The effects of tetramycin + matrine, tetramycin, and matrine on the quality of kiwifruit fruits are displayed in Table 6. Compared to control, tetramycin + matrine could significantly (*p* < 0.05) increase vitamin C, soluble sugar, soluble solid and dry matter of kiwifruit fruits, as well as decrease their titratable acidity. Simultaneously, vitamin C, soluble sugar, soluble solid and dry matter of kiwifruit fruits treated by tetramycin + matrine were a little more than those of tetramycin or matrine alone. However, the quality improvement of kiwifruit fruits treated by tetramycin or matrine alone was not obvious, and they could only significantly (*p* < 0.05) increase dry matter content of fruits compared with control. These findings show that matrine used together with tetramycin could effectively enhance kiwifruit fruit quality, and tetramycin and matrine should have a notably synergistic effect in improving the quality of kiwifruit fruits.

### 3.5. Effects of Tetramycin and Matrine on Amino Acids of Kiwifruit Fruits

The effects of tetramycin + matrine, tetramycin, and matrine on kiwifruit amino acids are displayed in Table 7. Total amino acids of fruits treated by tetramycin + matrine, tetramycin, and matrine was higher than that of control. Compared to control, the sweet, flavor, bitter, essential, nonessential and total amino acids of fruits treated by tetramycin + matrine were significantly (*p* < 0.05) higher than those of control. Simultaneously, tetramycin + matrine could also significantly (*p* < 0.05) enhance the sweet, flavor, essential, nonessential and total amino acids of fruits compared with tetramycin treatment. Meanwhile, tetramycin + matrine also could significantly (*p* < 0.05) enhance the flavor, bitter, essential and nonessential amino acids of fruits compared with matrine treatment. Aromatic amino acids of fruits in four treatments showed no significant (*p* < 0.05) differences. Moreover, compared to control, tetramycin could only significantly (*p* < 0.05) increase bitter amino acids of kiwifruit fruits, while matrine could only significantly (*p* < 0.05) increase sweet, flavor and nonessential amino acids. These results reveal that the promoted effects of fruit amino acids by tetramycin + matrine were superior to those of tetramycin or matrine alone.

## 4. Discussion

Tetramycin is a natural product derived from microorganisms, while matrine is also a natural product derived from plants, and both of them have many prominent advantages, including easy degradation, no residue poisoning and no environmental pollution [35,37]. Our previous studies found that tetramycin exhibited superior antimicrobial activity against various kiwifruit pathogens [3,27]. Simultaneously, previous findings have demonstrated that matrine could effectively inhibit the mycelia biosynthesis of *Cochliobolus miyabeanus*, *Rhizoctonia solani*, *Fusarium moniliforme*, *Alternaria brassicae* and *Cercospora sorghi* [34,35]. The results here exhibit that 0.5% matrine AS also had an outstanding toxicity against *B. dothidea* RF-1 and *Phomopsis* sp. RF-2 with EC_50_ values of 0.442 and 0.332 mg kg^−1^, which were higher by 42.43 and 77.85, 191.73 and 116.03, 212.49 and 172.30, and 3081.70 and 737.73 folds compared to 1.0% osthole EW, 80% ethylicin EC, 0.5% physcion AS and 0.5% berberine AS, respectively. This work expanded the antimicrobial spectrum of matrine. Additionally, although 0.3% eugenol SL had a relatively superior toxicity against *B. dothidea* RF-1 or *Phomopsis* sp. RF-2, its toxicity to dominant pathogen *B. dothidea* was inferior to that of 0.5% matrine AS. Furthermore, 0.5% matrine AS and 0.3% tetramycin AS had the same dosage form; hence, they were optimized as combined fungicides for controlling soft rot disease in kiwifruit.

Combined application of fungicides consisting of two or more active components may present antagonistic, additive or synergistic interaction [35,38]. In our previous study, we also found that co-application of tetramycin and chitosan was more effective than tetramycin or chitosan alone in controlling soft rot disease of kiwifruit and enhancing the disease resistance, growth, quality and aroma of kiwifruit fruits [12], while the combined application of tetramycin and matrine in controlling plant diseases has not been reported. In this study, the control effect of tetramycin + matrine against soft rot disease was 82.68%, which was significantly (*p* < 0.05) higher than 75.19% of tetramycin and significantly (*p* < 0.01) higher than 68.50% of matrine. This suggests that tetramycin and matrine had a notably synergetic effect in the control of soft rot disease of kiwifruit. This synergistic interaction was a combined action of many factors, and it was probably derived from the superior antimicrobial activities and complementary action modes of tetramycin and matrine, which effectively decreased aggressiveness of *B. dothidea* and *Phomopsis* sp. of soft rot pathogens. Synergistic application of fungicides can not only expand their antimicrobial spectrum, enhance fungicide efficiency, reduce fungicide amount and decrease control cost, they can also play a key role in overcoming and delaying fungicide resistance. Tetramycin is a polyene antibiotic mixture, and combined with matrine, it can effectively prevent the resistance development of pathogens [27,39].

Phenolics and flavonoids are two important secondary metabolites in systemic resistance of plants. Soluble protein and MDA are closely related to plant disease resistance [40]. In our previous report, Wang et al. [12,27] found that tetramycin could effectively increase phenolics and flavonoids of kiwifruit fruits. In this work, matrine used together with tetramycin effectively improved phenolics, flavonoids and soluble protein of fruits and inhibited their MDA, whereas these effects of matrine or tetramycin alone were dissatisfactory. SOD, POD, PPO, and PAL are closely associated with plant disease resistance [40]. Zhong et al. [39] found that tetramycin could stimulate plant disease resistance by enhancing PPO, PAL and POD activities. Wang et al. [12,27] also indicate that tetramycin notably promoted SOD and PPO activities of kiwifruit fruits. Similarly, the present results show that matrine used together with tetramycin notably improved SOD, POD, PPO, and PAL activities of kiwifruit fruits, while these effects of matrine or tetramycin alone were unsatisfactory. These results emphasize that the co-application of tetramycin and matrine was more helpful in improving the disease resistance of kiwifruit, and an obviously synergetic interaction of tetramycin and matrine was available.

The health of kiwifruit fruits during storage determines its fruit quality and commodity. Wang et al. [12] indicated that chitosan + tetramycin effectively enhanced the growth and quality of kiwifruit. In this study, tetramycin + matrine significantly (*p* < 0.05) enhanced volume, weight, vitamin C, soluble solid, soluble sugar and dry matter of kiwifruit and reduced their titratable acidity. However, the development and quality improvements of kiwifruit fruits treated by tetramycin or matrine alone was not obvious. These findings show that matrine used together with tetramycin could effectively enhance kiwifruit fruit development and quality, and tetramycin and matrine should have a notably synergistic interaction. These notable effects were probably derived from the dual action of tetracycin and matrine, of which their co-application could protect kiwifruit from pathogenic infection and induce disease resistance in kiwifruit fruits. The closer the amino acid composition of foods is to that of human proteins, the higher its nutritional value [3]. According to the amino acid model of protein nutritional value proposed by the WHO and FAO, it is suggested that the essential amino acids with superior quality account for about 40% of total amino acids, and the ratio of essential amino acids to nonessential amino acids is more than 0.6 [3,41]. In this study, the aforementioned percentages and ratios of kiwifruit fruits treated by tetramycin + matrine, tetramycin, matrine and control were 31.40% and 0.52, 31.46% and 0.53, 30.89% and 0.51, as well as 31.15% and 0.52, respectively. These results illustrate that the protein nutritional value of kiwifruit fruits treated by tetramycin + matrine was closer to the ideal mode value compared to matrine and control treatments. Simultaneously, tetramycin + matrine could significantly (*p* < 0.05) increase sweet, flavor, bitter, essential, nonessential and total amino acids of kiwifruit fruits, and the promoted effects of fruit amino acids by tetramycin + matrine were superior to those of tetramycin or matrine alone. These findings highlight that matrine is an effective adjuvant of tetramycin in enhancing its improvement for kiwifruit quality.

At present, natural products are preferred by consumers and are increasingly used as popular alternatives or complementary approaches to fungicides for plant disease management [13,14,42]. Tetramycin and matrine not only have many prominent advantages, such as easy degradation, no residue poisoning and no environmental pollution, etc., they have also been widely used in the medicine and agriculture fields [18,29,35,37]. Meanwhile, the field application concentration of 0.3% tetramycin AS (5000-fold dilution liquid) + 0.5% matrine AS (1000-fold dilution liquid) is low, and the safe interval (August 1 to September 28, 58 days) and soft ripening (more than 20 days) periods of kiwifruit fruits were also long. Thus, the food safety risks caused by tetramycin or matrine are almost nonexistent. This study highlights that the co-application of tetramycin and matrine can be used as a feasible candidate approach for controlling soft rot disease of kiwifruit.

## 5. Conclusions

In conclusion, tetramycin and matrine displayed excellent toxicity activities against *B. dothidea* and *Phomopsis* sp. The combination of tetramycin and matrine effectively controlled soft rot disease of kiwifruit and reliably promoted the contents of total phenolics, total flavonoids and soluble protein of kiwifruit fruits and decreased their MDA, as well as notably enhanced their SOD, POD, PPO, and PAL activities. Moreover, the combination of tetramycin and matrine could effectively improve the development, quality and amino acids of kiwifruit fruits. This study highlights that the combination of tetramycin and matrine can be used as a feasible candidate approach for controlling soft rot disease of kiwifruit.

## Figures and Tables

**Table 1:** Information of tetramycin and botanical fungicides.

Fungicides	Dosage Forms	Manufactures	Manufacture Sites
0.3% Tetramycin	Aqueous solutions (AS)	Microke Biological Engineering Co. Ltd.	Liaoning, China
0.5% Matrine	AS	Xinghe Crop Science and Technology Co. Ltd.	Shandong, China
0.3% Eugenol	Soluble liquid (SL)	Baoding Yada Chemical Co. Ltd.	Hebei, China
1.0% Osthole	Emulsion in water (EW)	Suke Agrochemical Co. Ltd.	Jiangsu, China
80% Ethylicin	Emulsifiable concentrate (EC)	Kebang Chemical Co. Ltd.	Henan, China
0.5% Physcion	AS	Qingyuanbao Biological Technology Co. Ltd.	Neimenggu, China
0.5% Berberine	AS	Wante Biochemical Co. Ltd.	Hebei, China

**Table 2:** The physical and chemical characteristics of planting soils in the kiwifruit orchard.

Parameters	Content	Parameters	Content
Organic matter	35.63 g kg^−1^	Exchangeable calcium	18.09 cmol kg^−1^
Total nitrogen	1.43 g kg^−1^	Exchangeable magnesium	312.67 mg kg^−1^
Total phosphorus	1.71 g kg^−1^	Available zinc	0.81 mg kg^−1^
Total potassium	1.15 g kg^−1^	Available iron	31.54 mg kg^−1^
Alkali-hydrolyzable nitrogen	98.75 mg kg^−1^	Available manganese	18.68 mg kg^−1^
Available phosphorus	7.31 mg kg^−1^	Available boron	0.15 mg kg^−1^
Available potassium	1.83 mg kg^−1^	pH	5.93

**Table 3:** Toxicities of tetramycin and botanical fungicides against *B. dothidea* and *Phomopsis* sp.

Pathogens	Fungicides	Regression Equation	Determination Coefficient (*R*^2^)	EC_50_ (mg kg^−1^)
*B. Dothidea* RF-1	0.3% Tetramycin AS	*y* = 6.076 + 1.251*x*	0.996	0.143
	0.5% Matrine AS	*y* = 5.422 + 1.191*x*	0.978	0.442
	0.3% Eugenol SL	*y* = 5.365 + 2.180*x*	0.991	0.680
	1.0% Osthole EW	*y* = 4.201 + 0.628*x*	0.981	18.752
	80% Ethylicin EC	*y* = 2.065 + 1.522*x*	0.993	84.745
	0.5% Physcion AS	*y* = 2.858 + 1.086*x*	0.971	93.919
	0.5% Berberine AS	*y* = 4.100 + 0.287*x*	0.943	1362.110
*Phomopsis* sp. RF-2	0.3% Tetramycin AS	*y* = 1.151 + 9.360*x*	0.997	0.094
	0.5% Matrine AS	*y* = 5.925 + 1.882*x*	0.923	0.332
	0.3% Eugenol SL	*y* = 5.710 + 1.360*x*	0.997	0.301
	1.0% Osthole EW	*y* = 3.178 + 1.290*x*	0.996	25.847
	80% Ethylicin EC	*y* = 3.139 + 1.174*x*	0.953	38.521
	0.5% Physcion AS	*y* = 4.117 + 0.502*x*	0.991	57.205
	0.5% Berberine AS	*y* = 3.533 + 0.614*x*	0.991	244.928

Note: *y* and *x* indicate the inhibition rate and fungicide concentration, respectively.

**Table 4:** The control effects of tetramycin and matrine on soft rot diseases of kiwifruit.

Treatments	Incidence Rate of Disease Fruits (%)	Control Effects (%)
Tetramycin + Matrine Tetramycin	9.78 ± 1.39 ^cC^	82.68 ± 2.46 ^aA^
	14.00 ± 2.00 ^bBC^	75.19 ± 3.54 ^bAB^
Matrine	17.78 ± 2.14 ^bB^	68.50 ± 3.80 ^cB^
Control	56.44 ± 3.01 ^aA^	

Note Values indicate the mean ± SD (*n* = 3). Different capital and small letters in the same column show significant differences at 1% (*p* < 0.01) and 5% (*p* < 0.05) levels, respectively.

**Table 5:** The effects of tetramycin and matrine on the development of kiwifruit.

Treatments	Diameter (mm)			Fruit Shape Index	Single Fruit Volume (cm^3^)	Single Fruit Weight (g)
	Longitudinal	Transverse	Lateral			
Tetramycin + Matrine Tetramycin	76.89 ± 0.31 ^a^	52.98 ± 0.50 ^a^	42.64 ± 0.24 ^a^	1.61 ± 0.00 ^a^	72.72 ± 1.00 ^a^	91.81 ± 0.59 ^a^
	76.68 ± 0.22 ^a^	52.68 ± 0.43 ^a^	41.86 ± 0.52 ^a^	1.62 ± 0.01 ^a^	70.79 ± 0.81 ^ab^	90.42 ± 0.86 ^ab^
Matrine	76.14 ± 0.46 ^a^	52.05 ± 0.51 ^a^	41.97 ± 0.28 ^a^	1.62 ± 0.01 ^a^	69.64 ± 1.09 ^ab^	89.72 ± 0.73 ^bc^
Control	76.10 ± 0.56 ^a^	52.03 ± 0.30 ^a^	41.59 ± 0.24 ^a^	1.63 ± 0.01 ^a^	68.95 ± 1.12 ^b^	88.93 ± 1.06 ^c^

Note: Values indicate the mean ± SD (*n* = 3). Different small letters in the same column show significant differences at 5% level (*p* < 0.05).

**Table 6:** The effects of tetramycin and matrine on quality of kiwifruit.

Treatments	Vitamin C (g kg^−1^)	Total Soluble Sugar (%)	Soluble Solid (%)	Dry Matter (%)	Titratable Acidity (%)
Tetramycin + Matrine Tetramycin	1.90 ± 0.02 ^a^	12.62 ± 0.06 ^a^	15.50 ± 0.10 ^a^	19.68 ± 0.11 ^a^	1.05 ± 0.01 ^b^
	1.87 ± 0.02 ^ab^	12.40 ± 0.10 ^ab^	15.27 ± 0.15 ^a^	19.37 ± 0.19 ^ab^	1.12 ± 0.04 ^a^
Matrine	1.87 ± 0.01 ^ab^	12.61 ± 0.05 ^a^	15.17 ± 0.15 ^a^	19.34 ± 0.17 ^ab^	1.09 ± 0.02 ^ab^
Control	1.85 ± 0.01 ^b^	12.10 ± 0.08 ^b^	14.70 ± 0.10 ^b^	18.98 ± 0.14 ^b^	1.11 ± 0.03 ^a^

Note: Values indicate the mean ± SD (*n* = 3). Different small letters in the same column show significant differences at 5% level (*p* < 0.05).

**Table 7:** The effects of tetramycin and matrine on amino acids of kiwifruit fruits.

Amino Acids (g kg^−1^)	Tetramycin + Matrine	Tetramycin	Matrine	Control
Aspartic	0.89	0.83	0.86	0.83
Glutamate	1.85	1.84	1.85	1.79
Cystine	0.97	0.93	0.96	0.97
Serine	0.80	0.76	0.77	0.76
Glycine	0.77	0.65	0.76	0.75
Histidine	0.69	0.68	0.68	0.66
Arginine	1.44	1.38	1.41	1.35
Threonine	0.45	0.48	0.48	0.47
Alanine	0.76	0.68	0.74	0.67
Proline	1.25	1.28	1.26	1.29
Tyrosine	0.67	0.68	0.68	0.67
Valine	0.65	0.60	0.65	0.64
Methionine	0.57	0.63	0.57	0.58
Isoleucine	0.62	0.60	0.58	0.58
Leucine	0.65	0.59	0.57	0.58
Phenylalanine	0.74	0.70	0.72	0.68
Lysine	0.94	0.85	0.88	0.87
Sweet amino acids	4.72 ± 0.01 ^a^	4.53 ± 0.04 ^b^	4.69 ± 0.05 ^a^	4.60 ± 0.04 ^b^
Flavor amino acids	3.68 ± 0.03 ^a^	3.51 ± 0.03 ^c^	3.58 ± 0.01 ^b^	3.49 ± 0.01 ^c^
Bitter amino acids	3.92 ± 0.08 ^a^	3.81 ± 0.04 ^ab^	3.78 ± 0.01 ^bc^	3.73 ± 0.04 ^c^
Aromatic amino acids	2.37 ± 0.03 ^a^	2.31 ± 0.08 ^a^	2.36 ± 0.03 ^a^	2.32 ± 0.01 ^a^
Essential amino acids	4.61 ± 0.07 ^a^	4.45 ± 0.07 ^b^	4.45 ± 0.03 ^b^	4.41 ± 0.03 ^b^
Nonessential amino acids	8.83 ± 0.04 ^a^	8.42 ± 0.04 ^c^	8.70 ± 0.04 ^b^	8.45 ± 0.04 ^c^
Total amino acids	14.69 ± 0.05 ^a^	14.16 ± 0.10 ^b^	14.42 ± 0.08 ^ab^	14.15 ± 0.04 ^b^

Note: Values indicate the mean ± SD (*n* = 3). Different small letters in the same line show significant differences at 5% level (*p* < 0.05).

## Data Availability

The datasets during or analyzed during the current study are available from the corresponding author upon reasonable request.
